# Environmental safety of electronic and construction and demolition waste based geopolymer breakwaters under simulated marine leaching

**DOI:** 10.1038/s41598-026-58190-x

**Published:** 2026-06-23

**Authors:** K. K. D. A. Wijesekara, Iacopo Carnacina, Monower Sadique, U. Dave, M. M. A. B. Abdullah

**Affiliations:** 1 Built Environment and Sustainable Technologies (BEST) Research Institute, School of Engineering and Built Environment, Liverpool John Moores University, Byrom Street, Liverpool, L3 3AF UK; 2 Department of Civil Engineering, Nirma University, S G Highway, Ahmedabad, Gujarat 382481 India; 3 Faculty of Chemical Engineering & Technology, Universiti Malaysia Perlis (UniMAP), D/A Pejabat Pos Besar, P.O. Box 77, 01000 Kangar, Perlis Malaysia

**Keywords:** Geopolymer concrete, Leaching mechanism, Metal ion, Breakwaters, Recycled silicate, Chemistry, Environmental sciences

## Abstract

This study presents the evaluation of the environmental performance of a geopolymer based breakwater using a novel dual-waste valorisation approach that combines electronic waste (e-waste) derived sodium silicate from cathode ray tube glass (CRT) as a geopolymer activator, and construction and demolition waste (C&DW) aggregates. It will systematically investigate the leaching of major (Ca, Si, Al, Mg, Na) and trace/heavy metals (Cr, As, Zn, Fe, Pb, Ni, Cd, Cu) under simulated marine exposure. Geopolymer blocks incorporating e-waste derived sodium silicate and C&DW aggregates were compared with ordinary Portland cement (OPC) concrete and commercial silicate-based geopolymers. Standard dynamic surface leaching tests were performed in deionized water (DIW) and seawater (SW) for 64 days, with extrapolated predictions for a 50-year service life. Cumulative release of major ions during 64-day immersion ranged from 3.5 to 700 gm^−2^, while trace/heavy metals remained within 30–150 mgm^−2^. Extrapolation over 50 years indicated that all trace/heavy metals, including Pb, Cd, Ni, Cu, Mn, As, Cr, Zn, and Fe, would remain below 1 gm^−2^, while abundant elements such as Al, Mg, Si, Na, and Ca would remain below 1 kgm^−2^ in seawater. Higher Na (in GEO-RSiA-NA) and Ca (in NC-NA and GEO-RSiA-RA) releases were observed in DIW compared to SW, confirming its more aggressive leaching environment. Overall, trace/heavy metal concentrations remained well below Dutch regulatory thresholds, confirming the environmental safety of geopolymer breakwaters. These findings demonstrate the feasibility of recycled silicate–aggregate geopolymers as a sustainable breakwater material with reduced carbon footprint and controlled environmental impact. Further field validation under real hydrodynamic forces is recommended.

**Supplementary Information:**

The online version contains supplementary material available at https://doi.org/10.1038/s41598-026-58190-x.

## Introduction

The search for sustainable and eco-friendly construction materials has become a critical priority in the field of civil engineering due to the growing environmental concerns associated with the construction industry, particularly the high carbon footprint of Portland cement production. In recent years, there has been a significant interest in geopolymer concrete as an alternative material that offers both ecological and economic benefits. Geopolymers are inorganic polymers synthesized through the alkaline activation of industrial by-products such as fly ash, slag, and other waste materials. Geopolymerization effectively immobilizes a wide range of contaminants both chemically and physically within aluminosilicate precursors, highlighting its potential for environmental stabilization applications^1^. Furthermore, the incorporation of industrial by-products into geopolymer concrete offers an innovative solution to managing waste materials while improving the overall sustainability of construction practices^2–5^.

Among the various non-structural applications, breakwaters are vital coastal structures designed to protect shorelines from erosion, mitigate the impact of waves, and safeguard the stability of the coastline. Breakwaters are constantly exposed to harsh marine environments, including seawater, high salinity, temperature fluctuations, and physical forces from waves. These aggressive conditions pose significant challenges to the long-term stability and durability of construction materials used in coastal infrastructure^6–8^. As climate change continues to exacerbate the frequency and intensity of extreme weather events, and in parallel with softer engineering solutions, the demand for durable and sustainable coastal protection structures, such as breakwaters, has increased. The use of alternative, environmentally friendly materials, such as geopolymer concrete, has gained attention as a potential solution for reducing the ecological footprint of such structures while maintaining their performance and longevity^9^. While geopolymer concrete presents a promising alternative to traditional Portland cement-based materials, its activators, such as sodium hydroxide (NaOH) and sodium silicate (Na_2_SiO_3_), remain carbon-intensive due to their energy-demanding production processes^10^. In particular, the manufacturing of sodium silicate requires high-temperature processing, contributing to significant CO_2_ emissions and partially offsetting the environmental benefits of geopolymers^11^.

To mitigate this issue, alternative activators derived from recycled materials have been explored, including silicate sources recovered from waste products such as cathode ray tube (CRT) glass. CRT glass, which contains high silica concentrations (60–65% SiO_2_), can be processed into a viable precursor for geopolymer activators, reducing dependence on synthetic sodium silicate and promoting a circular economy approach^12^. Studies have shown that silicate solutions extracted from CRT waste glass can effectively activate geopolymerization, demonstrating comparable or even superior mechanical and durability properties to conventional activators^13^. By integrating recycled silicate sources, such as those derived from CRT glass, the sustainability of geopolymer concrete can be significantly enhanced, making it a more environmentally friendly option for breakwaters and other marine infrastructure applications. This approach not only reduces carbon emissions but also helps in managing electronic waste, aligning with global sustainability efforts.

However, while geopolymer concrete shows promise in many civil engineering applications, its long-term behaviour in aggressive environments, particularly in terms of leaching properties, remains insufficiently explored. Pradhan et al.^14^ underscored that the environmental durability of geopolymer materials remains one of the least-explored aspects of sustainable construction, especially concerning the potential release of soluble ions into aquatic environments. Leaching refers to the process by which ions and other harmful constituents are dissolved from the material and transported into the surrounding environment, potentially leading to contamination of water bodies and affecting the structural integrity of concrete over time^15^. The release of harmful ions, such as alkali metals, heavy metals, and other pollutants, can compromise both the environmental quality of the surrounding ecosystem and the long-term service life of concrete structures exposed to seawater^16^. This is of particular concern in the case of breakwater structures, which are directly exposed to seawater and face dynamic and corrosive conditions that may accelerate degradation processes^17^.

In recent years, the environmental impact of cement-based materials, particularly their leaching behaviour, has attracted significant attention due to their potential consequences on water quality and ecosystem health. These works have highlighted the importance of investigating the leaching mechanisms of both conventional and alternative cementitious materials in marine environments. For example, a series of studies have demonstrated that leaching from concrete structures, including Portland cement and geopolymer-based materials, is influenced by factors such as water chemistry, pH, and salinity, particularly in coastal applications^18^. Exposure to seawater can increase the rate of ion leaching due to the high ionic content and alkalinity of marine environments, potentially accelerating the release of harmful substances and compromising structural durability. However, studies have shown that fly ash-based geopolymers maintain strength after seawater exposure^19^ and suffer only minimal mass loss (~ 3–5%) compared to OPC concretes^20^. These studies emphasize the necessity of developing geopolymer-based alternatives with minimized leaching characteristics, as their long-term durability and environmental compatibility remain essential considerations for marine applications such as breakwaters.

While the formulation, mechanical performance, and durability of this waste based geopolymer mixes were established in our earlier study^21^, that work did not assess their environmental performance under marine exposure. The present study specifically investigates the leaching behaviour, release mechanisms, and long-term extrapolated metal ion release of these optimized geopolymer blocks under standardized dynamic surface leaching conditions in deionized water and simulated seawater. Therefore, this paper provides new knowledge on environmental safety and service life related release behaviour, which was outside the scope of the previous mechanical study. Despite the potential advantages, the leaching of geopolymer concrete, especially when composed of waste-based precursors such as fly ash and slag, has not been sufficiently studied in the context of marine applications and coastal protection. This knowledge gap is critical because breakwaters and other coastal structures are exposed to a complex combination of physical and chemical stressors, which may influence the leaching process in ways that are not yet fully understood.

Investigations into the release of harmful substances from hardened concrete have been conducted primarily in a few countries, including the Netherlands and Germany. In Germany, only specific materials are tested, with approval required from the German Institute for Building Technology. The first standard, NEN 7345, was developed in the Netherlands in 1995 and revised in 2004 as NEN 7375. The German equivalent was introduced by the German Committee for Reinforced Concrete in 2005 as a guideline. A harmonized European technical specification, the “DSLT,” was published in 2014 and corresponding British standard in 2023 (BS EN 16637‑2:2023). Predicting the long-term release of harmful substances is crucial when assessing how construction materials behave over their service life in a building. To make such predictions through extrapolation, it’s essential to understand the release mechanism (i.e. diffusion or dissolution) involved. The release mechanism of a substance can often be determined from the results of the DSL test.

Accordingly, this study aims to evaluate the long-term release of both major and trace metal ions from waste-based geopolymer breakwater blocks in comparison with conventional OPC concrete. The investigation applies standardized dynamic surface leaching tests in deionized water and seawater to simulate realistic marine environments, followed by 50-year extrapolation based on BS EN 16637-2: 2023. By focusing on the leachate kinetics/mechanisms and environmental safety of optimized geopolymer systems, this work complements previous mechanical and durability research and provides a critical step toward the qualification of sustainable geopolymer materials for coastal infrastructure applications.

To date, no studies have investigated the combined use of e-waste-derived activators and recycled aggregates in marine applications. This dual-waste valorisation strategy, transforming both electronic waste and construction demolition waste into functional marine infrastructure, addresses two major waste streams simultaneously while reducing the carbon footprint associated with virgin material production. The novelty of this work lies in demonstrating that such waste-based geopolymer systems not only meets the mechanical performance requirements^21^, but also the stringent environmental safety standards for coastal applications.

## Materials and methods

### Materials

**Fig. 1:**
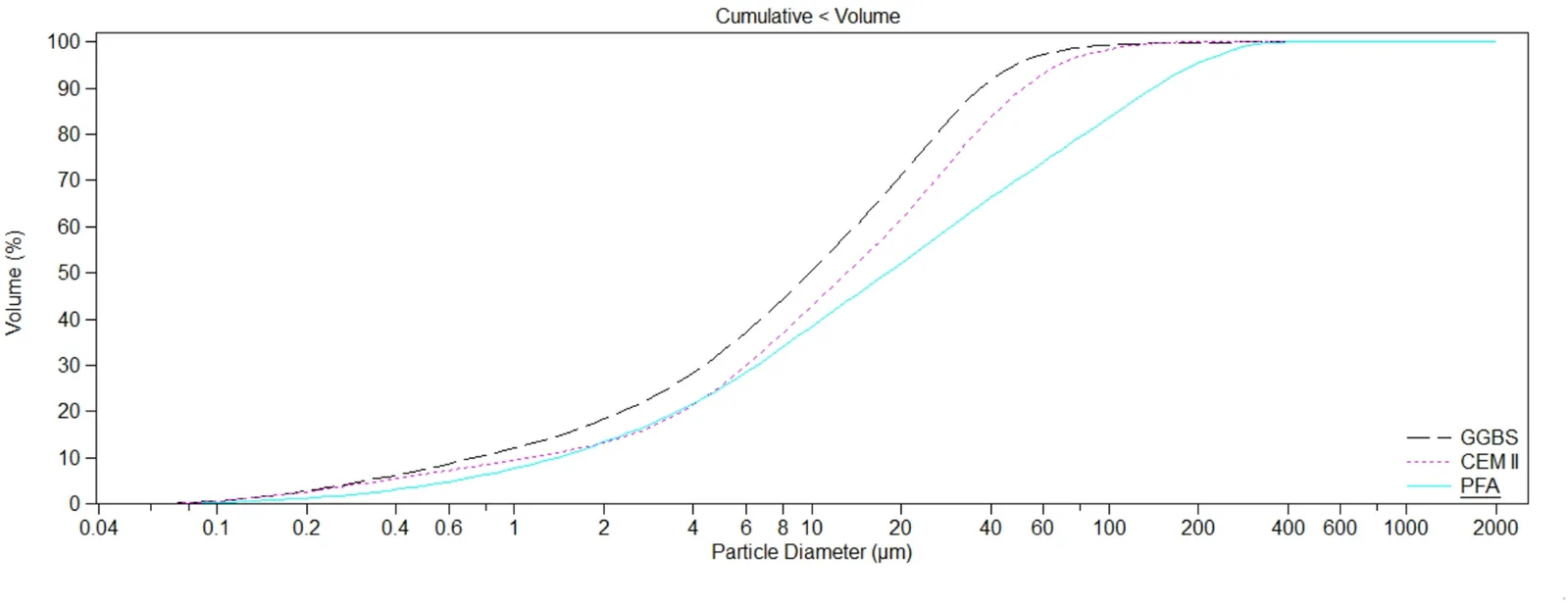
Particle size distribution (cumulative volume) of binders.

A geopolymer where an equal proportion of fly ash and granulated blast furnace slag (GGBS) were used as silica and alumina-containing solid precursor sources respectively activated with NaOH, (NH) and Sodium silicate, Na_2_SiO_3_ (NS). For the control mix, CEM II 32.5N as per BS EN 197–1:2011 (British Standard Institution^22^) was used. The elemental composition of binders and the silicates used for Geoblocks is shown in Tables 1 and 2, and the particle size distributions (PSD) are presented in Fig. 1.

**Table 1:** Elemental composition (%) of binders from EDX.

	Al	Si	P	S	Cl	K	Ca	Ti	V	Cr	Mn	Fe	Ni	Cu	Zn	As	Pb
PFA	15.54	38.86	0.64	1.52	0.04	5.56	9.03	2.16	0.12	0.05	0.17	24.59	0.08	0.07	0.20	0.04	0.07
GGBS	6.26	15.67	–	1.80	0.00	0.91	71.70	1.39	0.05	–	0.54	0.87	–	–	–	–	–
CEM II	2.2	26.7	0.9	-	–	0.6	64.1	1.3	–	–	0.2	2.4	-	–	–	–	–

**Table 2:** Elemental composition of commercial and recycled silicate.

	O	Na	Si
Commercial silicate	56.2	16.1	27.7
Recycled silicate	59.3	21.02	19.68

Natural aggregates, NA: 10 mm and 20 mm aggregates that accord with the British Standard BS EN 12620:2002+A1:2008 (British Standard Institution^23^) have been used. The quantity of 10 mm and 20 mm was adjusted to get a similar particle size distribution to that of recycled aggregates. Fine natural dried sand with a size under 600 µm was used as natural fine aggregate (NFA).

**Fig. 2:**
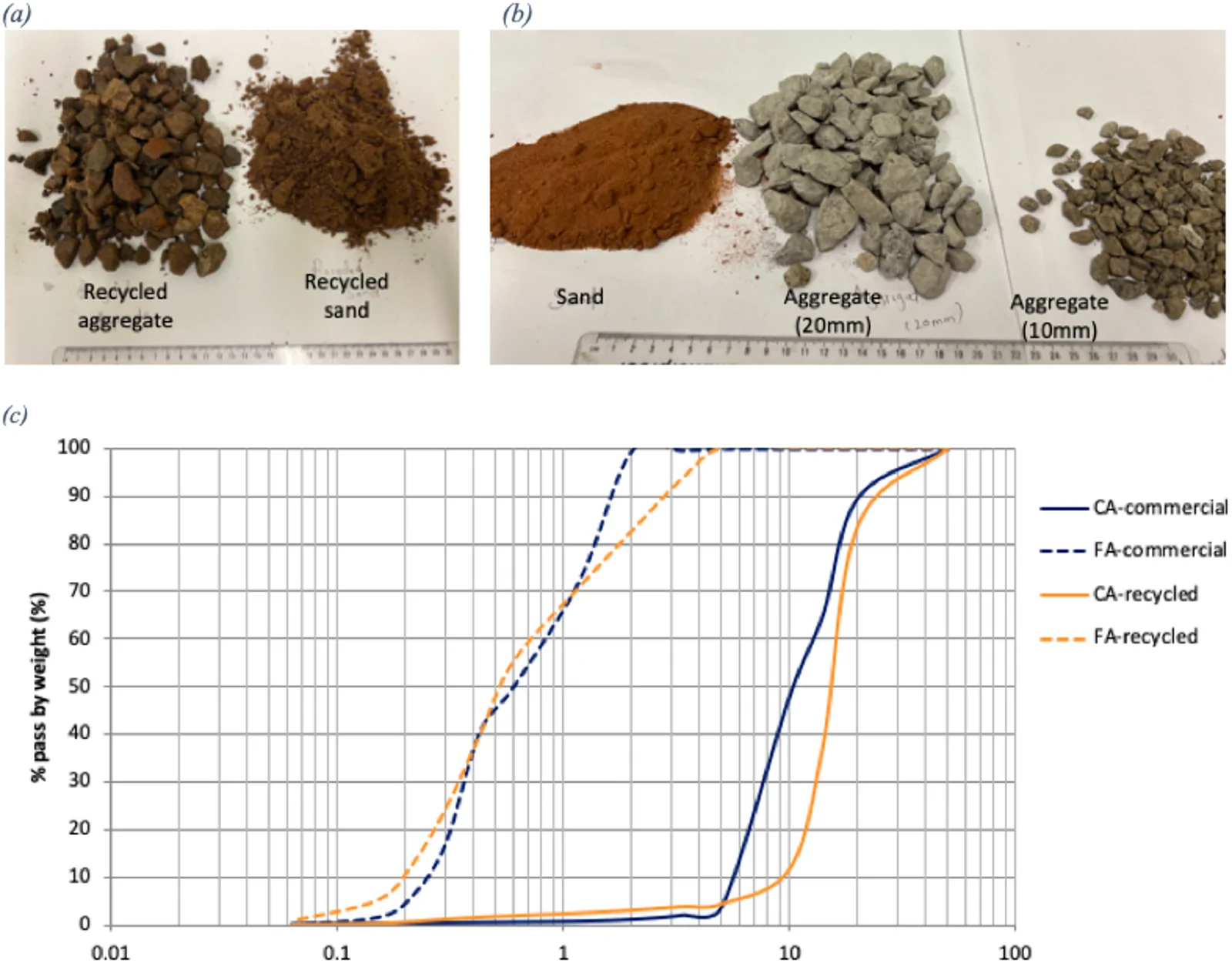
Images of (**a**) RA (**b**) NA (scale in mm) (**c**) particle size distribution of RA and NA.

Recycled aggregates (RA): Recycled coarse aggregate (CA) and recycled fine aggregates (RFA) were supplied by a local waste management organization that processes construction and demolition waste (C&DW) which was a combination of different materials (ceramic, plastic, metal, clay, glass, concrete, or stones), densities, shape, roughness and porosities. The PSD was determined by dry sieving according to the BS EN 933-1 (British Standards Institutions^24^). Three individual determinations were conducted on separate test oven-dried portions (105 ± 2 °C until mass stabilization) of RA. The range of sieves employed covered up to 34 mm. The amount of 10 mm aggregates was such to adjust the particle size distribution (PSD) to that of RA (Fig. 2).

The recycled sodium silicate used in this study represents a breakthrough in e-waste valorisation. Extracted from end-of-life CRT monitor glass through proprietary processing by industrial partners, this activator diverts hazardous electronic waste from landfills while providing equivalent or superior activation performance compared to commercial sodium silicate. The combination of CRT-derived activator with recycled C&DW aggregates creates a fully waste-based geopolymer system, a waste vaporization process not previously reported for marine applications.

The activators used represent the alkaline source that increases the pH of the chemical reaction and eases the dissolution^10^. For this research, the liquid activators 4 M sodium hydroxide, NaOH (NH) solution from Fisher Scientific and sodium silicate, Na_2_SiO_3_ (NS) Technical, Solution, d = 1.5 from Fisher Chemical was used to prepare the alkali activator. Sodium silicate, Na_2_SiO_3_ and 4 M Sodium hydroxide, NaOH were used in a proportion 2:1. The development and optimization of this activator–precursor mix design has been described in detail in^21^. While mechanical and durability analysis of geopolymer is well known and streamlined, the environmental quality of emerging material is often neglected, and a holistic characterization of these emerging material needs to be carried out to conform with ecological and sustainability standards. This research will therefore fill this gap by manly focuses on the environmental performance of Geoblocks thorough the associated leachate characteristics and dynamics,

In the following, the Geoblocks will also be benchmarked against standard geopolymer concrete using natural virgin aggregate (NA) and RA was cast in addition to a control mix where 100% cement with only NA was used with a binder-to-aggregate ratio of 1:3.

### Methods

#### Sample preparation

Effect of leaching behaviour when using recycled aggregate and recycled silicate (RSiA) was assessed by comparison of cubes (100 mm × 100 mm × 100 mm) produced in mix designs; Normal concrete with NA (NC-NA), Geopolymer mix using commercial silicate and NA (GEO-CsiA-NA). Geopolymer mix using recycled silicate and NA (GEO-RsiA-NA), Geopolymer mix using recycled silicate and RA (GEO-RsiA-RA).

All blocks (Normal concrete and Geoblocks) were produced with liquid: binder ratio 1:2 and binder: aggregate ratio 1:3. NC-NA was produced using 100% CEM-II (binder) and water (liquid) while Geoblocks were produced using FA: GGBS 40:60 (binder) and Na_2_SiO_3_: NaOH (liquid) ratio 2:1 (C40-2111). This mix design was based on prior optimization published in another study^21^ based on preliminary mechanical strength and tests on mortar.

Dry precursors and aggregates were first mixed for 5 min , after which the pre-prepared alkaline activator was added and mixing continued for further 5 min to achieve a homogeneous mixture. The fresh mix was poured into 100 × 100 × 100 mm moulds in two layers with vibration/compaction after each layer. Specimens were demoulded after 24 h and then cured in air at room temperature for 28 days prior to testing.

#### Horizontal dynamic surface leaching test

The horizontal dynamic surface leaching test (DSLT) was conducted in accordance with BS EN 16637‑2:2023 (British Standard Institution^25^,) both in deionised (DI) water and 3% sodium chloride (NaCl) solution to mimic the NaCl concentration of sea water (SW) since the application of Geoblocks are for breakwaters for coastal protection. Two blocks from each composition NC-NA, GEO-CsiA-NA, GEO-RsiA-NA, and GEO-RsiA-RA were submerged in 9.6 L where the water volume (L) divided by the sample surface area (A), L/A ratio was 80 L/m^2^: For 100 mm cubes, the geometric surface area used for DSLT calculations was 0.06 m^2^ per specimen, assuming all six faces were exposed. The liquid to exposed area ratio was therefore maintained at 80 L/m^2^ in accordance with BS EN 16637-2:2023. The test species were submerged in the tank in such a way that space in between two test specimens and distance between walls of vessel and specimen are > 20 mm. The eluates were sampled at fixed intervals of 0.25, 1, 2.25, 4, 9, 16, 36, and 64 days changing the liquid (with same amount) after measuring pH and conductivity on each day.

#### Analysis of water and suspended solids from slake durability test

The analysis of water and suspended solids from the slake durability test is conducted to assess the potential release of materials under dynamic wave action in coastal applications. Since breakwaters and other marine infrastructure are constantly exposed to wave forces, understanding the material’s resistance to degradation and the extent of leaching or particle release is crucial for evaluating its long-term durability and environmental impact. The slake durability test was performed in accordance with ASTM D4644-1 for all compositions of test materials in DIW and SW. Ten pieces each weighing between 40 and 60 g, and their total initial mass was recorded. These specimens were placed in a wire mesh drum which was partially submerged in a trough filled water. The drum was rotated at 20 rpm for a duration of 10 min. Following the rotation, the specimens were removed, oven-dried, and the total mass retained in the drum was measured to calculate the % weight loss. Effluent water from the test was collected in sample tube to analyse by ICP-OES and suspended solids during slake test were dried and analysed via EDX.

#### Sample analysis by ICP-OES

The elements to be analysed were chosen based on nature and elemental composition of materials used in the mix (Table 1). Each of the sampled eluates were filtered by 0.25 µm filters and were acidified to make them in 2 vol% nitric acid. Sample eluates in sea water were diluted 10 times prior analysis to reduce total dissolved content. Concentrations of Na, Ca, Mg, Al, Si, Fe, As, Cd, Cr, Cu, Mn, Ni, Pb, Zn were then measured using Inductively Coupled Plasma Optical Emission Spectrometry (ICP-OES) 5110, Agilent. Analysis was conducted with three replicates for each sample, utilizing both axial and radial measurements to ensure accuracy and reliability. The seawater eluates were diluted 10 times to reduce the total dissolved content and minimize matrix effects during ICP-OES analysis to ensure more accurate quantification of target elements by preventing signal suppression or interference from high salt concentrations.

## Results

The release of metal ions to the water from NC-NA, GEO-CsiA-NA, GEO-RsiA-NA, and GEO-RsiA-RA by DSLT (in both DI water and sea water) and after slake test in DIW and SW are presented with the view of understanding how the blocks would leach in static and dynamic contact by tides.

### pH value and electrical conductivity

The pH values and electrical conductivity (EC) measurements of eluates in deionized water (DIW) and seawater (SW) provide valuable insights into the behaviour of geopolymer materials under simulated marine conditions. The observed pH ranges, 7.57–10.8 in DIW and 6.54–10.67 in SW, are consistent with findings in existing literature, where geopolymer leachates typically exhibit alkaline pH levels^10,11^. This alkalinity is primarily influenced by the physicochemical properties of the solid precursors, the dosage of alkaline activators, and the extent of geopolymerization^26^.

Electrical conductivity values in DIW eluates ranged from 181 to 1412 µS/cm, while those in SW eluates varied between 28.6 and 50.7 mS/cm. The higher EC values in SW are attributed to the elevated salinity of seawater, which enhances the ionic conductivity of the solution. These conductivity measurements align with findings from Istuque et al.^27^, who demonstrated that the ionic conductivity of geopolymer composites is closely influenced by precursor composition and curing conditions, as revealed through impedance spectroscopy.

**Fig. 3:**
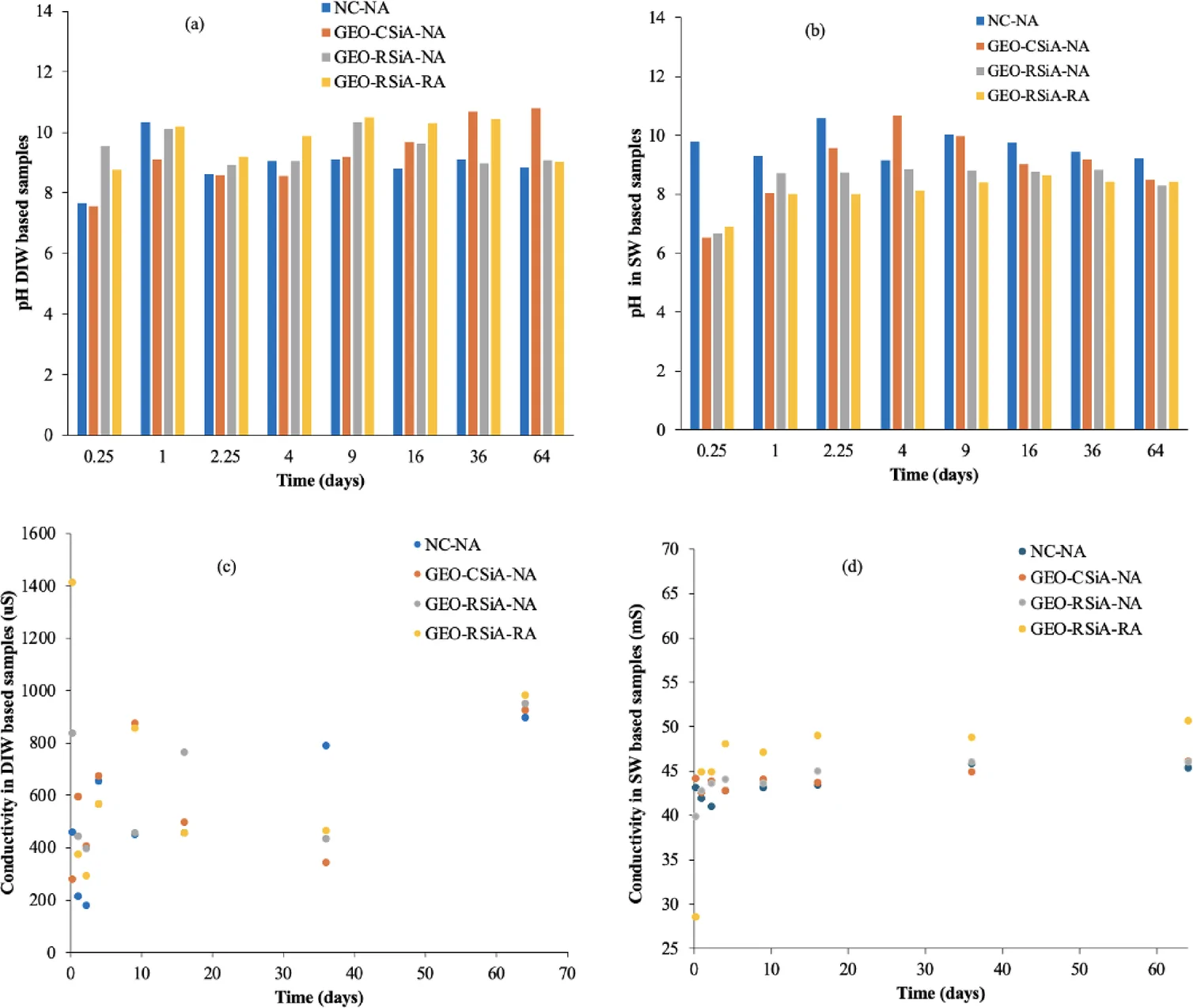
pH of eluates in (**a**) DIW and (**b**) SW and conductivity of eluates in (**d**) DIW and (**e**) SW.

From an environmental perspective, pH and EC are important indicators of the interaction between geopolymer materials and surrounding water, although they should not be interpreted as direct predictors of field impact. In standardised leaching tests, the chemistry of the eluent strongly influences the measured response. Deionized water represents a conservative and more aggressive medium due to its low buffering capacity, which enhances the concentration gradient between the pore solution and the external liquid, potentially overestimating release compared to mineralised waters. In contrast, saline waters provide higher ionic strength and buffering, where precipitation, complexation, and re-adsorption processes may reduce the dissolved ion concentration. Similar observations on alkaline geopolymer leachates and their influence on surrounding aqueous chemistry have been reported by Sun & Vollpracht^28^, while Habert et al.^11^ highlighted the importance of ionic interactions and environmental assessment of geopolymer systems. Therefore, pH and EC should be interpreted together with cumulative elemental release data when assessing the environmental compatibility of geopolymer materials for coastal applications (Fig. 3).

### Leaching of major components

**Fig. 4:**
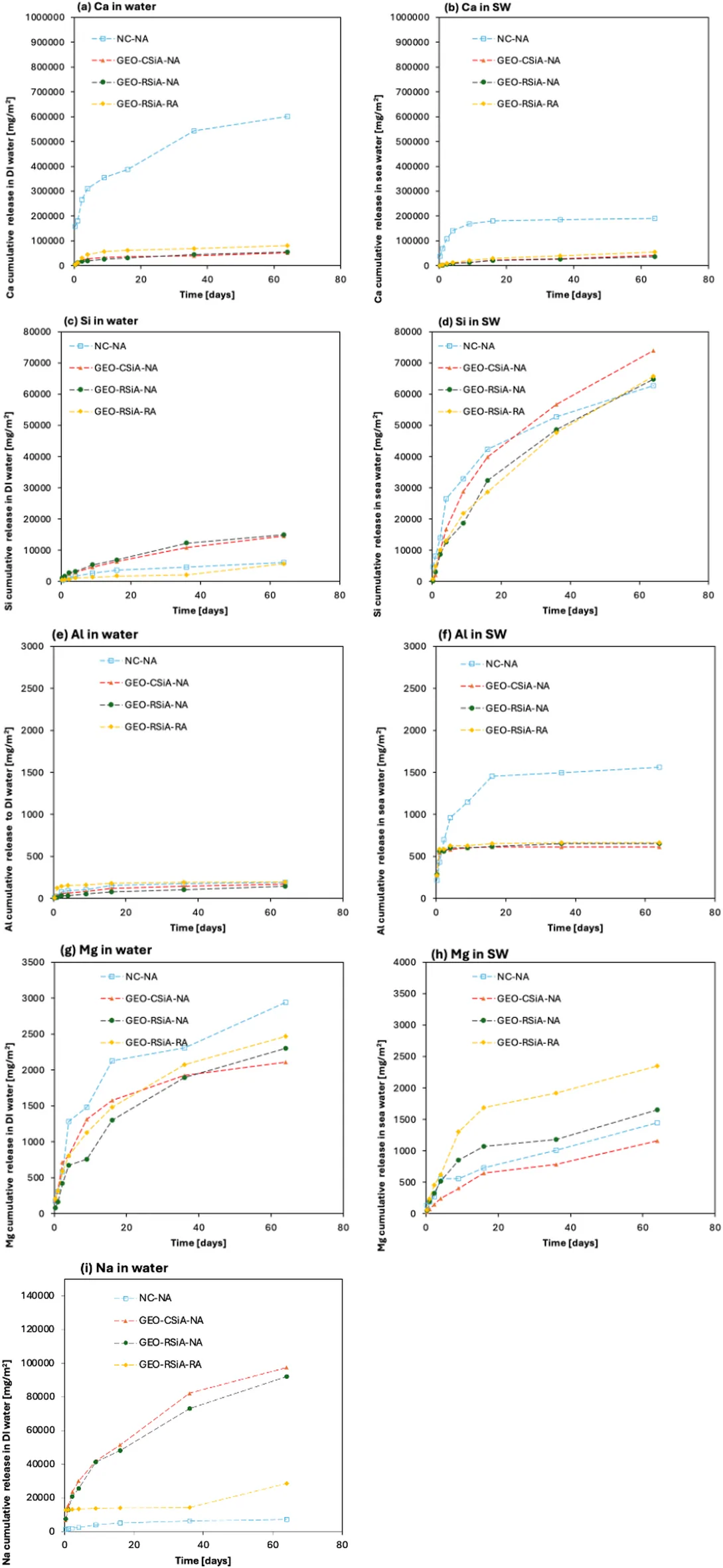
Cumulative release of major elements, Ca, Si, Al, Mg and Na to DI water and sea water from concrete blocks.

**Fig. 5:**
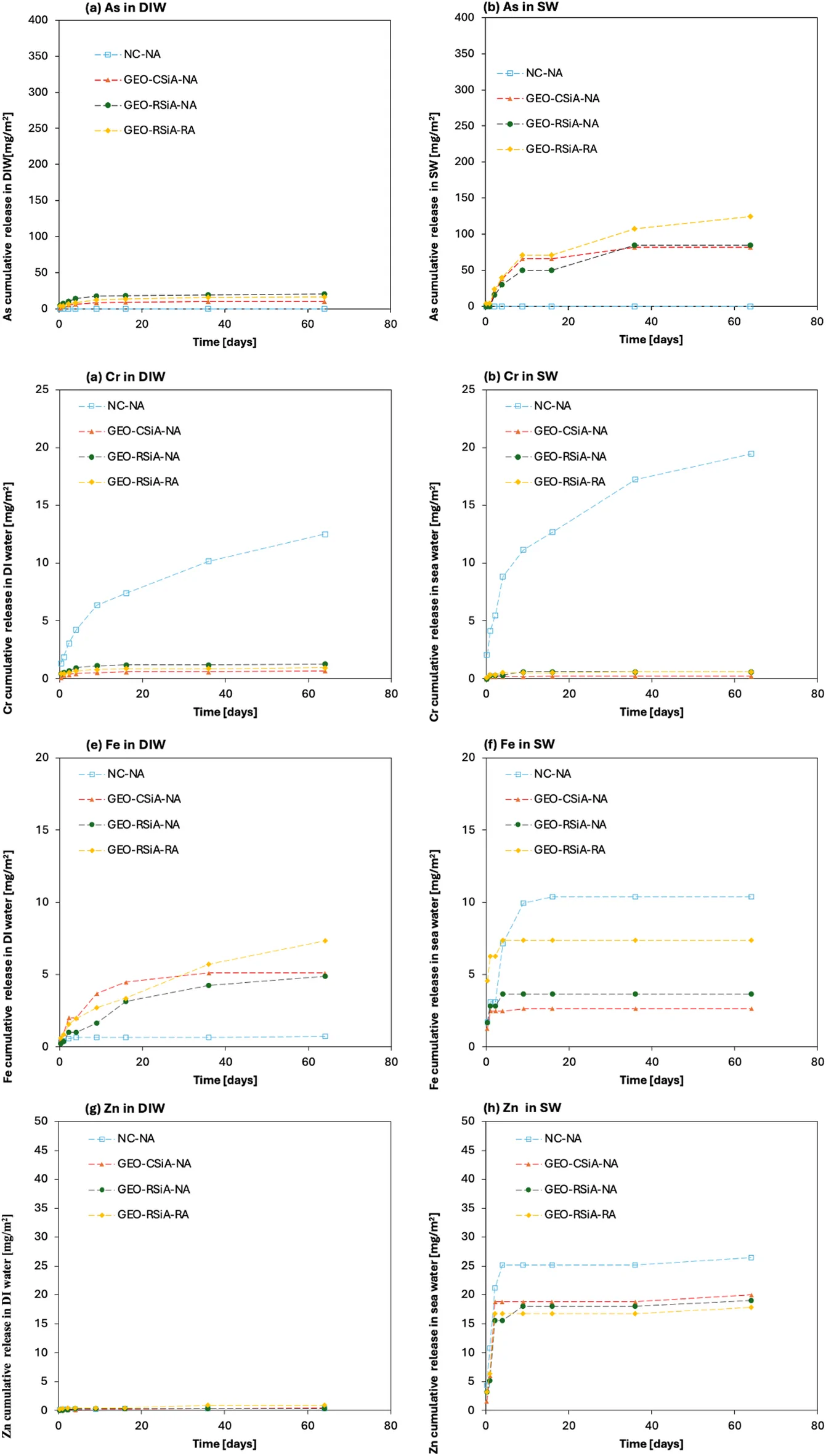
Cumulative release of As, Cr, Fe and Zn to DI water and sea water from concrete blocks.

The ICP-OES results were analysed using calibration curves with an R^2^ > 0.995. Cumulative release of major elements (present in the constituents of blocks) to SW and DIW were calculated based on results from ICP-OES and plotted against each time interval to understand the leaching kinetics. For major elements with the range of cumulative release of 3500–700,000 mgm^−2^ over 64 days, Ca, Si, Al, Mg and Na are presented in Fig. 4 while for other elements with the range of cumulative release of 30–150 mgm^−2^ over 64 days, Zn, Fe, Cr and As are presented in Fig. 5.

The concentrations for Cd, Cu, Mn, Ni and Pb were below the thresholds defined by German and Netherland thresholds^29,30^ and below the limit of quantification (LOQ) (which is defined by LOQ = 3 × LOD) and therefore was not presented. Ca levels in NC-NA are comparably higher than the Geoblocks which could be attributed to high Ca content in CEM-II (64%) compared to geopolymer binder, FA: GGBS = 40:60 (47%). This difference is also mechanistic. In OPC systems, Ca is mainly associated with reactive Ca-rich phases that are more susceptible to dissolution in low-ionic-strength solutions. In geopolymer mixes, the aluminosilicate/C-A-S-H gel network provides greater chemical stability, reducing ion mobility through chemical binding and physical encapsulation. Release of Ca was less significant in SW eluates than in DIW eluates. This is consistent with the lower thermodynamic driving force for dissolution in saline media and/or with secondary precipitation reactions in seawater, where dissolved Ca may be removed from solution as calcium carbonate and calcium sulfate, thereby lowering the concentration measured in filtered eluates^31^. The use of DIW therefore creates a deliberately aggressive leaching environment and may overestimate release relative to field marine exposure^32^. Accordingly, the saline results are considered more representative of the intended breakwater application.

**Fig. 6:**
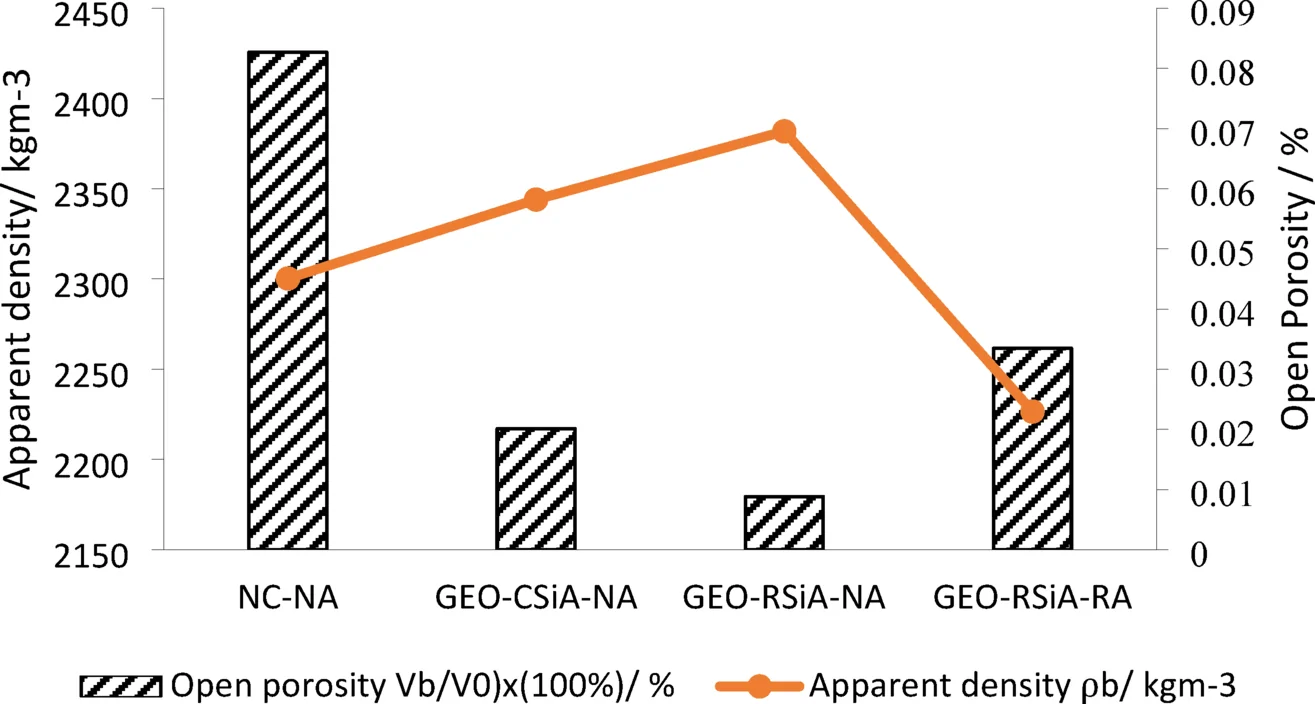
Open porosity and apparent density of NC-NA, GEO-CSiA-NA, GEO-RSiA-NA and GEO-RSiA-RA after 28 days curing (data elaborated from^21^).

Geopolymer concrete showed less release of Mg and Cr than normal concrete, which is consistent with restrained transport through a denser matrix and the lower solubility of geopolymer binders compared with OPC^33^. The correlation with Fig. 6 is also meaningful for the present dataset: NC-NA had the highest open porosity (0.08%) and showed the highest release of Ca, Mg, and Cr, whereas GEO-RSiA-NA had the lowest open porosity (0.01%) and among the lowest cumulative releases for most diffusion-controlled species^21^. Lower pore connectivity reduces liquid ingress and limits diffusive transport from the interior to the exposed surface, explaining why the denser geopolymer blocks generally exhibited lower release for these elements. By contrast, Na and Fe behaved differently: their higher release from geopolymers is better explained by source chemistry than by porosity alone, since Na is introduced during alkali activation and Fe is more abundant in fly ash (24.59%) than in OPC (2.4%) (Refer Table 1). Thus, leaching behaviour is governed by both transport properties and elemental speciation/availability, not by porosity alone.

Wijesekara et al.^21^ have also demonstrated that the lower density of recycled silicate and aggregate-based mixes, compared to commercial silicate and aggregate-based counterparts, is attributed to the presence of less dense aggregates. However, when these aggregates are encapsulated within the binder matrix in larger Geoblocks, the influence of porosity on elemental release is significantly reduced. Although geopolymer blocks exhibited a higher silicon composition due to the presence of silicate-rich binders and GGBS/FA compared to OPC-based mixes, the release of Si, Al, As, and Zn remained nearly identical across all formulations. This was attributed to their incorporation into insoluble phases within the stable aluminosilicate framework^16,34^.

The above interpretation is consistent with published microstructural studies linking denser geopolymer matrices and stabilising gel phases to lower heavy-metal mobility. SEM–EDS would therefore be a valuable next step to verify pore connectivity, phase distribution, and secondary precipitates after leaching.

### Release of trace/heavy metals

Cumulative release of heavy metals and trace metals to DIW and SW over 64 days are compared against German and Dutch thresholds in Table 3. The Dutch and German criteria were used here as established benchmark values for the release of hazardous substances from construction products. They are therefore useful reference points for comparing relative release potential between formulations and with previous studies on monolithic cementitious and geopolymer materials, but they are not marine-specific ecological quality standards. Accordingly, compliance with these values supports a low release potential under standardised conditions, but it should not be interpreted as a complete site-specific risk assessment for coastal ecosystems, which would additionally require seawater exposure scenarios, dilution considerations, and ideally ecotoxicity testing.

**Table 3:** Cumulative release of heavy/trace metal ions over 64 days in SW and DIW.

Element	Mix design	Cumulative release over 64 days			
		Leachate in DIW	Leachate in SW	German threshold	Dutch threshold
Pb	NC-NA	0.00	1.08	7.7	400
	GEO-CSiA-NA	0.00	1.24		
	GEO-RSiA-NA	0.00	2.48		
	GEO-RSiA-RA	0.00	1.36		
Cd	NC-NA	0.00	0.12	0.56	3.8
	GEO-CSiA-NA	0.00	0.12		
	GEO-RSiA-NA	0.00	0.08		
	GEO-RSiA-RA	0.00	0.12		
Ni	NC-NA	0.00	0.08	38.6	144
	GEO-CSiA-NA	0.00	0.00		
	GEO-RSiA-NA	0.00	0.24		
	GEO-RSiA-RA	0.00	0.80		
Cu	NC-NA	0.00	1.52	15.4	98
	GEO-CSiA-NA	0.12	0.56		
	GEO-RSiA-NA	0.28	0.52		
	GEO-RSiA-RA	1.92	1.00		
As	NC-NA	0.00	0.00	11	260
	GEO-CSiA-NA	10.28	8.20		
	GEO-RSiA-NA	20.20	8.44		
	GEO-RSiA-RA	16.60	12.44		
Cr	NC-NA	12.52	19.48	7.7	120
	GEO-CSiA-NA	0.68	0.24		
	GEO-RSiA-NA	1.28	0.60		
	GEO-RSiA-RA	0.96	0.60		
Zn	NC-NA	0.36	2.64	63.9	800
	GEO-CSiA-NA	0.44	2.00		
	GEO-RSiA-NA	0.32	1.90		
	GEO-RSiA-RA	0.96	1.79		

All the heavy/trace metal ions were below the Dutch threshold, indicating low release potential for the recycled-silicate and recycled-aggregate geopolymer blocks relative to established European construction-product benchmarks. Except for As in all mix designs and Cr in NC-NA, all other heavy/trace metal ions were also below the more stringent German threshold values. . Similar trends were reported by Mahfoud et al.^1^, who observed that geopolymerization effectively immobilizes As, Mo, Zn, Sb, Pb, Ni, Ba, Cr, and Cu, with leachate concentrations stabilizing after 16 days and remaining below the Dutch regulatory levels.These results indicate that substituting commercial silicate and virgin aggregate with recycled silicate and recycled aggregate did not measurably increase the release of environmentally relevant heavy/trace metals under the conditions tested. Furthermore, using geopolymer based mixes seems to be safer compared to cement based concrete with regards to Cr levels. Sun and Vollpracht^28^, studied the leaching of monolithic geopolymer mortars (fly ash, metakaolin and a commercial geopolymer) and all the heavy metals and trace element (except B, V and Mo) levels from geopolymer were below the German and Dutch thresholds and comparable to that of cementitious materials.

Finally, the comparable leaching performance between waste-based geopolymers (GEO-RSiA-RA) and commercial formulations (GEO-CSiA-NA) confirms that the use of e-waste-derived sodium silicate did not measurably increase hazardous metal release under the test conditions. This is particularly relevant because CRT glass is classified as hazardous waste due to the lead-rich funnel glass fraction. The present results support the conclusion that potentially harmful elements were effectively immobilised within the geopolymer matrix during the standardised leaching tests, with heavy-metal releases remaining below Dutch benchmark values. Combined with the use of recycled aggregates from C&DW, this approach demonstrates that two major waste streams, electronic waste and construction waste, can be valorised simultaneously into a low-release material suitable for further consideration in marine infrastructure.

### Identification of release mechanism

**Fig. 7:**
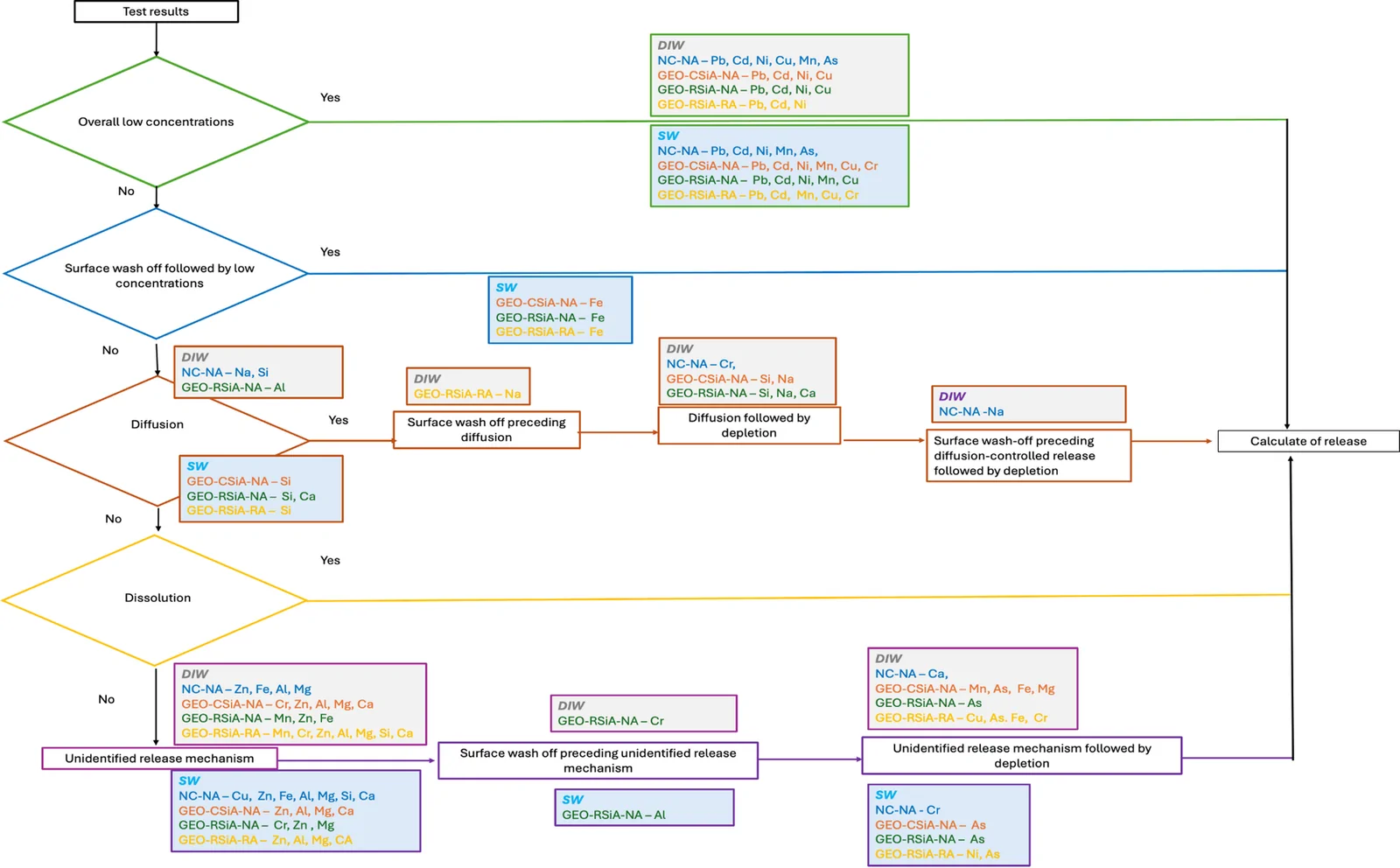
Release mechanisms of each metal ion in NC-NA – GEO - CSiA -NA, GEO–RSiA- NA in DIW and SW.

The concentrations of each metal ion released into DIW, along with the calculations used to determine their respective release mechanisms, are provided in Table S1 (Supplementary Material). These results offer insight into the dominant leaching processes governing metal ion mobility, aiding in the classification of their release behaviour in accordance with BS EN 16637-2:2023. The underlying leaching mechanisms of each metal ion are depicted in Fig. 7.

The release of chemicals from monolithic cementitious and geopolymer matrices is typically governed by surface wash-off, diffusion, and depletion processes, while some elements were categorised as exhibiting overall low concentrations or unidentified release mechanisms (BS EN 16637‑2:2023^35^;).In practical terms, diffusion-controlled release is associated with transport through the pore network and is therefore strongly influenced by porosity, pore connectivity, tortuosity, and degree of saturation. Surface wash-off reflects the rapid removal of readily soluble material located at or near the exposed surface and explains higher early-stage release for some elements. Depletion-controlled behaviour indicates exhaustion of a finite soluble reservoir, whereas unidentified or low-concentration classifications indicate that release was too small or too irregular to assign robustly.

Figure 7 presents the release classifications obtained for both DIW and SW, allowing the governing process to be related to the chemistry of each leachant rather than inferred from DIW alone. For marine infrastructure, this distinction is important because diffusion- and depletion-controlled species may contribute to low but persistent long-term emissions potentially influencing seawater chemistry over a 50-year design life^32,36^. Wash-off-dominated species are expected to stabilise rapidly after an initial conditioning period whereas this effect could be mitigated through pre-conditioning or rinsing of concrete blocks prior to deployment, thus reducing long-term environmental concerns^37,38^. potentially influencing seawater chemistry over a 50-year design life^32,36^. Materials However, if critical heavy metals or environmentally sensitive ions such as Pb, As, or Cr are involved, even low-concentration releases over decades could pose risks, particularly in ecologically sensitive coastal areas where bioaccumulation and sediment contamination may occur^39,40^. The generally lower release from geopolymer mixes is therefore consistent with both lower pore connectivity and stronger immobilisation within the aluminosilicate/C-A-S-H gel network.

Further modelling of the cumulative release over a 50-year period could help assess the total environmental impact and inform the selection of binder compositions that minimize long-term leaching. These insights are crucial for ensuring that geopolymer-based materials provide both structural durability and environmental sustainability in marine applications.

### Extrapolated release over 50 years

**Fig. 8:**
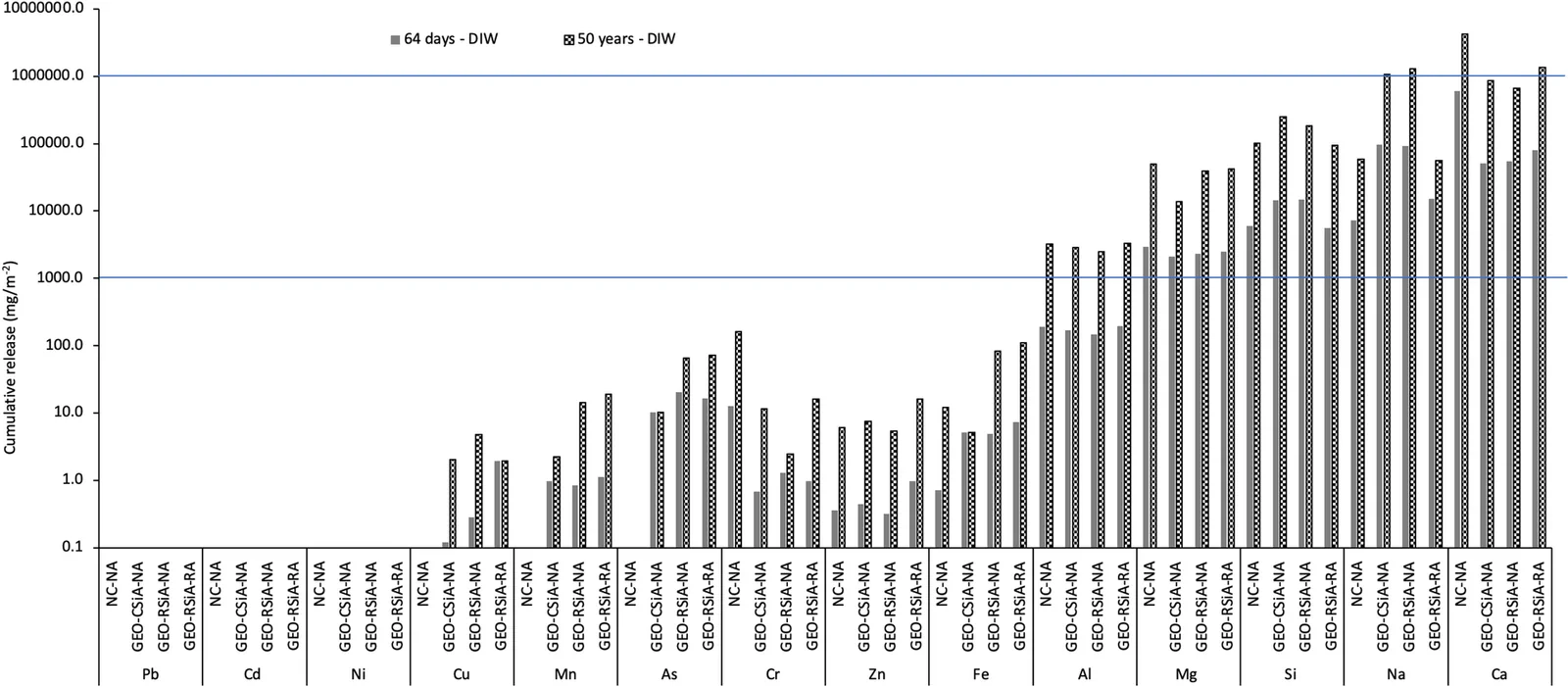
Cumulative reléase over 64 days and extrapolated reléase over 50 years of each metal ion in DIW.

**Fig. 9:**
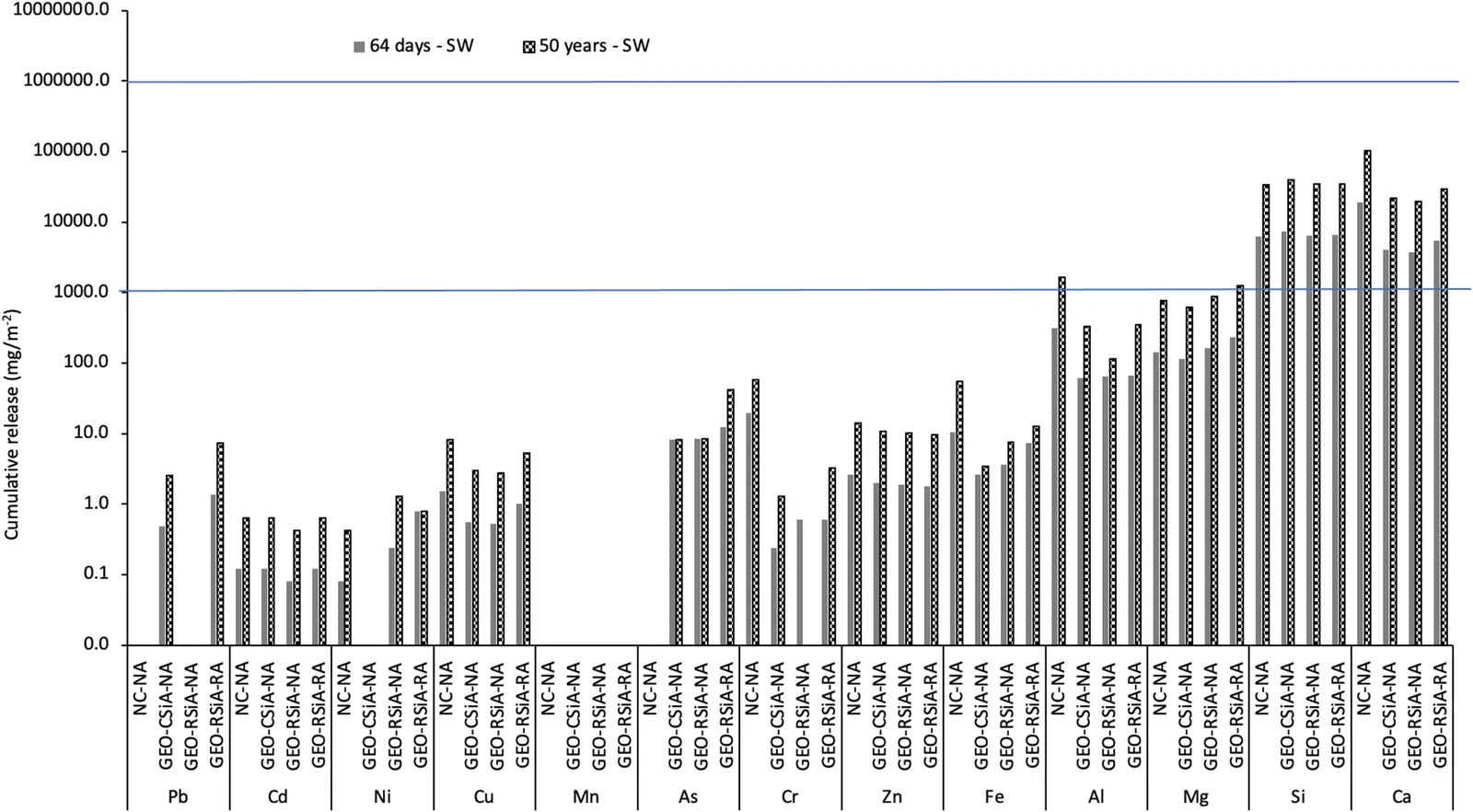
Cumulative reléase over 64 days and extrapolated reléase over 50 years of each metal ion in SW.

Based on the release mechanism of each metal ions depicted in Fig. 7, release over 50 years for each metal ion was calculated in accordance with BS EN 16,637‑2:2023 (British Standard Institution^25^,). The extrapolated values presented in Figs. 8 and 9 should therefore be interpreted as mechanism-based projections derived from the 64-day DSLT dataset, rather than exact predictions of 50-year field release under DIW and SW exposure conditions, respectively. The extrapolations were performed using the release classification identified for each respective leachant.The calculation is useful for ranking long-term release potential, but it assumes that the dominant release regime identified during the laboratory test remains valid over the projection period. Consequently, long-term changes in seawater chemistry, carbonation, biofouling, abrasion, and progressive microstructural alteration are not explicitly captured.

For all trace/heavy metal ions, including Pb, Cd, Ni, Cu, Mn, As, Cr, Zn, and Fe, the extrapolated release remained < 1 g m^−2^ of concrete over 50 years in both DIW and SW. The other metal ions present in greater abundance in the binder system, namely Al, Mg, Si, Na, and Ca, resulted in release < 1 kg m^−2^ of concrete over 50 years in SW, while Na (in GEO-RSiA-NA) and Ca (in NC-NA and GEO-RSiA-RA) were slightly above this level in DIW. This confirms the more aggressive nature of DIW due to its low ion concentration and lack of buffering^32^. The lower release of manganese (Mn) in SW compared with DIW is consistent with seawater oxidation chemistry, in which dissolved Mn(II) becomes less stable as pH and oxygen favour oxidation to insoluble oxide/hydroxide phases^32,40^. Competitive interactions with abundant seawater cations may further restrict Mn mobility at the solid–solution interface.

Additionally, competitive ion exchange with abundant seawater cations like Mg^2^⁺ and Ca^2^⁺ further restricts Mn leaching from the concrete matrix^41,42^.

### Effect of abrasion for leaching metal ions

**Fig. 10:**
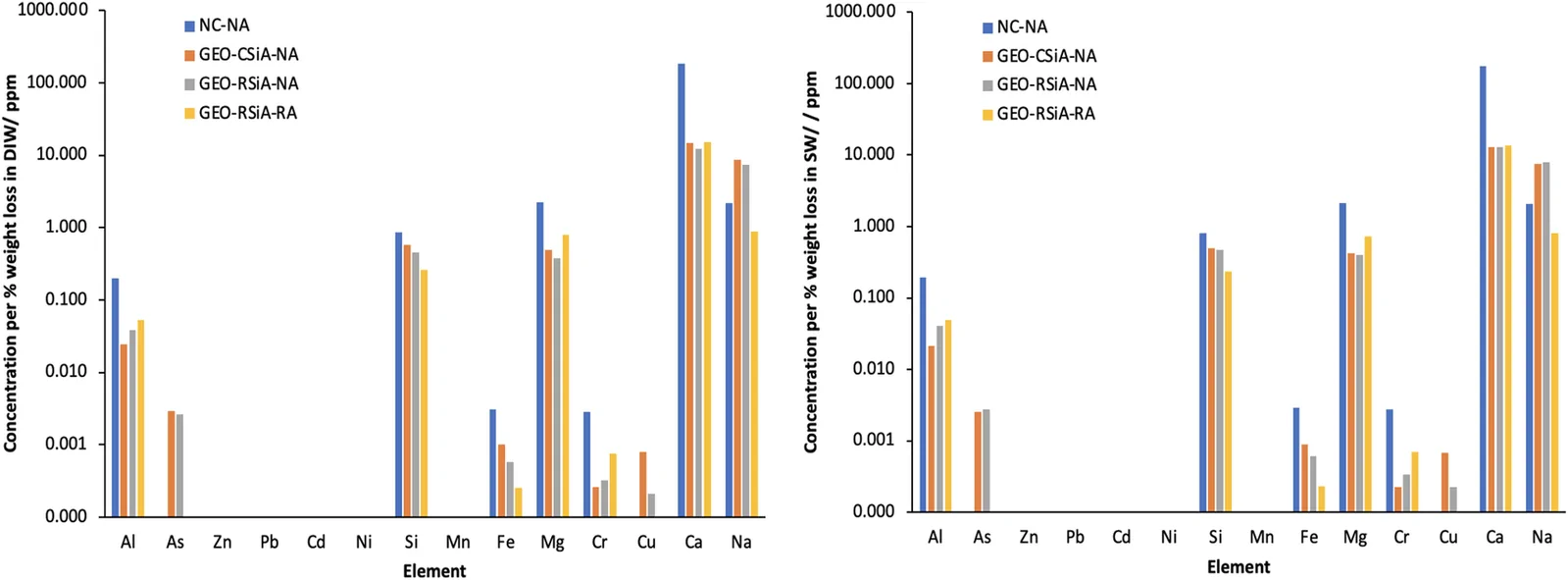
Release of metal ions per a percent of mass loss after slake durability test in DIW and SW.

Results from elemental analysis (by ICP-OES) of effluent water from the slake durability test in DIW and SW are presented per a percentage weight loss of material in Fig. 10. The slake durability results complement the DSLT by representing a more dynamic exposure condition in which abrasion, particle detachment, and repeated wetting can generate fresh surfaces. Trace/heavy metal ions Pb, Cd, and Ni were not detected in the eluates of any composition during the slake test, consistent with their negligible release in the 64-day DSLT results (Table 3). This indicates that mechanical abrasion did not expose a readily leachable reservoir of these hazardous trace metals. The absence of Cu and As in NC-NA but their presence in the geopolymer blocks, and the presence of Cr in NC-NA but not in geopolymer blocks, are trends that are also consistent with the static leaching results.

**Fig. 11:**
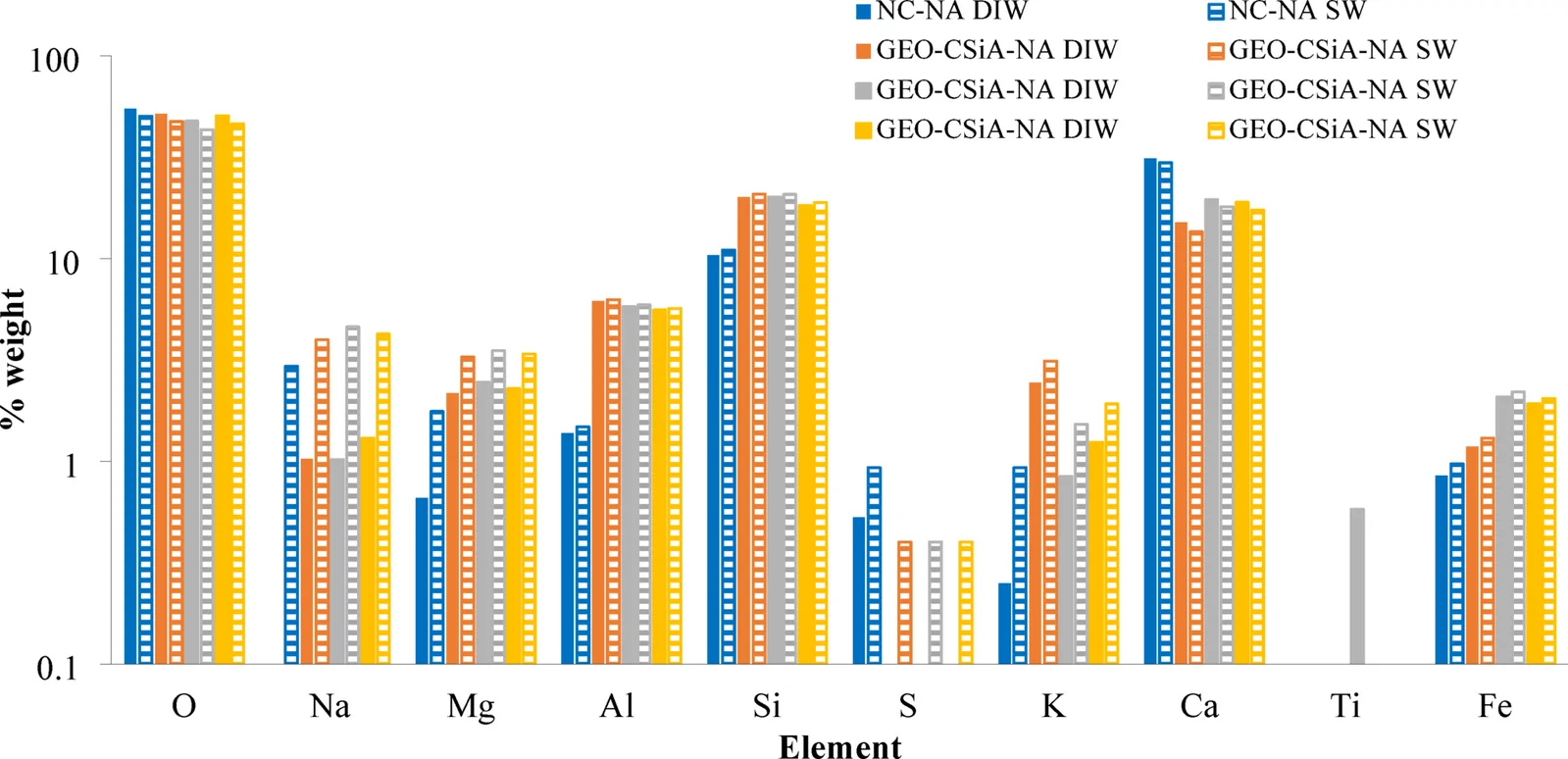
Elemental analysis of suspended solid after slake test in DIW and SW for all mix designs

Figure 11 shows the elemental composition of suspended solids resulted from slake durability test for both tested waters and all mix design.

Suspended solids released during abrasion were dominated by major matrix elements rather than hazardous trace metals, suggesting that abrasion primarily removed binder–aggregate fines rather than contaminant-enriched phases. While NC-NA exhibited some sulphur (S) release into the water following the slake durability test, the geopolymer blocks did not release S beyond what was already present in SW. Calcium concentrations in SW may again have been lowered by secondary precipitation as carbonate- or sulfate-bearing phases, while the higher ionic strength of SW may have stabilised or modified the release of Si and Al. In addition, chloride-rich conditions can influence Fe speciation and solubility, helping to explain the slight increase in Fe observed in SW compared with DIW. Overall, the abrasion results suggest that dynamic exposure did not substantially enhance the release of the most environmentally sensitive trace metals.

### Implications and limitations of the study

The present results support the environmental acceptability of the investigated waste-based geopolymer mixes under standardised laboratory leaching conditions. In particular, the combination of lower porosity, denser matrix structure, and effective immobilisation within aluminosilicate/C-A-S-H gels provides a plausible mechanistic explanation for why most trace and heavy metals remained at low release levels despite the use of recycled activator and recycled aggregate. However, several limitations should be recognised. First, the DSLT and slake tests provide controlled screening conditions rather than a full simulation of breakwater service, and the DIW case is intentionally conservative because deionized water exaggerates concentration gradients and lacks buffering capacity. Second, the saline leachant captures the chloride-rich and ionic-strength aspects of marine exposure but cannot fully reproduce the evolving chemistry, suspended particulate matter, biological activity, and hydrodynamics of natural coastal waters. Third, the Dutch and German threshold values are useful European benchmark criteria for release from construction products, but they were not developed specifically as marine ecological quality standards. Finally, the 50-year extrapolation is model-based and assumes persistence of the short-term release regime identified during the laboratory test.

Accordingly, the present results should be interpreted as strong evidence of low release potential rather than as a complete site-specific marine risk assessment. Future work should therefore combine long-term natural seawater exposure, hydrodynamic abrasion, ecotoxicity testing, and microstructural characterisation such as SEM–EDS to directly verify phase evolution, pore connectivity, and the localisation of retained or precipitated species.

## Conclusion

This study evaluated metal release from waste-based geopolymer breakwater blocks under standardised static and abrasion-related leaching conditions in deionised water and simulated seawater. Over the 64-day DSLT, cumulative release of major elements was on the order of 3.5–700 g m^−2^, whereas trace/heavy metals were much lower, typically 30–150 mg m^−2^, and remained comparable between the waste-based and commercial geopolymer formulations. Open porosity from the companion mechanical study remained low for the geopolymer mixes (0.01–0.03%) compared with NC-NA (0.08%), consistent with the lower release of several species, particularly Mg and Cr. Abrasion did not promote hazardous trace-metal release, as Pb, Cd and Ni remained below detection in the slake-test eluates for all mixes. Extrapolation of the DSLT data using the BS EN 16,637-2:2023 release-mechanism framework indicated 50-year releases of < 1 g m^−2^ for all trace/heavy metals in both DIW and SW, while major elements remained mostly below 1 kg m^−2^. These values should be interpreted as mechanism-based projections from the 64-day DIW and SW datasets rather than exact field predictions, because long-term changes in seawater chemistry and service exposure are not explicitly represented. Comparison with Dutch and German construction-product thresholds showed compliance with all Dutch limits and with most German limits, supporting their use as conservative first-tier screening benchmarks, although they are not marine-specific standards. Overall, replacing commercial silicate and natural aggregate with CRT-derived silicate and recycled C&DW aggregate did not increase environmentally relevant release and produced geopolymer units with leaching performance comparable to the reference materials. Further SEM–EDS based microstructural verification and long-term field exposure studies are now needed to confirm the immobilisation mechanisms under real coastal conditions.

## Supplementary Information

Supplementary Material1.

## Data Availability

All the data used in the present manuscript have been generated by the Authors. Any data will be provided upon request.

## Abbreviations

Al: Aluminium
As: Arsenic
ASTM: American Society for Testing and Materials
BS EN: British Standard European Norm
Ca: Calcium
Ca^2^⁺: Calcium Ion
CaCO_3_: Calcium Carbonate
CaSO_4_: Calcium Sulfate
CA: Coarse Aggregate
Cd: Cadmium
C&DW: Construction and Demolition Waste
CEM II: Portland Composite Cement (as per BS EN 197-1:2011)
CO_2_: Carbon Dioxide
Cr: Chromium
CRT: Cathode Ray Tube
Cu: Copper
DIW: Deionized Water
DSLT: Dynamic Surface Leaching Test
EC: Electrical Conductivity
FA: Fly Ash
Fe: Iron
GEO-CSiA-NA: Geopolymer Concrete with Commercial Silicate and Natural Aggregate
GEO-RSiA-NA: Geopolymer Concrete with Recycled Silicate and Natural Aggregate
GEO-RSiA-RA: Geopolymer Concrete with Recycled Silicate and Recycled Aggregate
GGBS: Ground Granulated Blast Furnace Slag
ICP-OES: Inductively Coupled Plasma Optical Emission Spectroscopy
K: Potassium
LOD: Limit of Detection
LOQ: Limit of Quantification
Mg: Magnesium
Mg^2^⁺: Magnesium Ion
Mn: Manganese
NA: Natural Aggregate
Na: Sodium
NaCl: Sodium Chloride
NaOH: Sodium Hydroxide
Na_2_SiO_3_: Sodium Silicate
NC-NA: Normal Concrete with Natural Aggregate
NH: Sodium Hydroxide (NaOH)
Ni: Nickel
NS: Sodium Silicate (Na_2_SiO_3_)
OPC: Ordinary Portland Cement
Pb: Lead
PFA: Pulverized Fly Ash
PSD: Particle Size Distribution
RA: Recycled Aggregate
S: Sulfur
Si: Silicon
SW: Seawater
Zn: Zinc

## References

[1] Mahfoud, E. et al. One-part geopolymer based on micronized sediments and fly ash mix: Mechanical, microstructural and leaching assessment. *J. Build. Eng.* **96**, 110426 (2024).

[2] Bakharev, T. Geopolymeric materials prepared using class F fly ash and elevated temperature curing. *Cem. Concr. Res.* **35**(6), 1224–1232 (2005).

[3] Fernandez-Jimenez, A., Garcia-Lodeiro, I. & Palomo, A. Durability of alkali-activated fly ash cementitious materials. *J. Mater. Sci.* **42**(9), 3055–3065 (2012).

[4] Kadhim, A., Sadique, M., Al-Mufti, R. & Hashim, K. Long-term performance of novel high-calcium one-part alkali-activated cement developed from thermally activated lime kiln dust. *J. Build. Eng.* **32**, 101766 (2020).

[5] Kadhim, A., Sadique, M., Al-Mufti, R. & Hashim, K. Developing one-part alkali-activated metakaolin/natural pozzolan binders using lime waste. *Adv. Cem. Res.* **33**(8), 342–356 (2021).

[6] Bernal, S. A. et al. Effect of binder content on the performance of alkali-activated slag concretes. *Cem. Concr. Res.* **41**(1), 1–8 (2011).

[7] Li, H. et al. Geopolymer composites for marine application: Structural properties and durability. *Cement Concrete Comp.* **152**, 105647 (2024).

[8] Shi, C., Krivenko, P. V. & Roy, D. M. *Alkali-Activated Cements and Concretes* 2nd edn. (CRC Press, 2019).

[9] Farooq, F., Jin, X. & Javed, M. F. Geopolymer concrete as sustainable material: A state-of-the-art review. *Constr. Build. Mater.* **306**, 124762 (2021).

[10] Provis, J. L. & Van Deventer, J. S. J. *Alkali-activated materials: State-of-the-Art Report, RILEM TC 224-AAM* (Springer, 2014).

[11] Habert, G., d’Espinose de Lacaillerie, J. B. & Roussel, N. An environmental evaluation of geopolymer based concrete production: Reviewing current research trends. *J. Clean. Prod.* **19**(11), 1229–1238 (2011).

[12] Górski, M. et al. Characteristics of metakaolinbased geopolymer incorporating cathoderaytube (CRT) glass aggregate. *Polymers* **13**(7), 1149 (2020).

[13] Grdić, Z., Topličić-Ćurčić, G., Radojević, Z., Despotović, I. & Miličević, I. Durability properties of concrete supplemented with recycled CRT glass as cementitious material. *Materials* **14**(1), 143 (2021). 10.3390/ma14164421 PMC8400815 34442942

[14] Pradhan, P., Dwibedy, S., Pradhan, M., Panda, S. & Panigrahi, S. K. Durability characteristics of geopolymer concrete—Progress and perspectives. *Journal of Building Engineering* **59**, 105100 (2022).

[15] Pacheco-Torgal, F., Ding, Y. & Palomo, A. Durability of alkali-activated binders. In *Durability of construction materials: Materials performance in coastal and marine environments* 1st edn (ed. Alexander, M. G.) 353–374 (CRC Press, 2013).

[16] Duxson, P., Provis, J. L., Lukey, G. C. & van Deventer, J. S. J. The role of inorganic polymer technology in the development of ‘green concrete’. *Cem. Concr. Res.* **37**(12), 1590–1597 (2007).

[17] Rangan, B. V. Geopolymer concrete. In *Geopolymer concrete: Properties and application* 1st edn (ed. Rangan, B. V.) 1–45 (University of Melbourne, 2008).

[18] Luukkonen, T. et al. Alkali-activated materials for water and wastewater treatment: A review. *Environ. Chem. Lett.* **17**(4), 1763–1779 (2019).

[19] Astutiningsih, W., Hardjito, D. & Wallah, S. E. Effect of seawater on compressive strength of fly ash based geopolymer concrete. *Int. J. Appl. Sci. Technol.* **2**(2), 1–7 (2010).

[20] Zaidi, F. H. A. et al. Geopolymer as underwater concreting material: A review. *Constr. Build. Mater.* **291**, 123276 (2021).

[21] Wijesekara, K. K. D. A., Sadique, M., Carnacina, I., Fielding, A. & Chronowska Bojczuk, G. Mechanical and durability analysis of geopolymer concrete made with recycled silicate activator for low carbon breakwaters. *Clean. Waste Syst.* **11**, 100322. 10.1016/j.clwas.2025.100322 (2025).

[22] British Standard Institution, BS EN 197-1:2011 - Cement. Part 1: Composition, specifi cations and conformity criteria for common cements. British Standard Institution, London, UK, 2011.

[23] British Standard Institution, BS EN 12620:2002+A1:2008 - Aggregates for concrete, British Standard Institution, London, UK, 2008.

[24] British Standards Institutions, BS EN 933-1:2012 - Tests for geometrical properties of aggregates. Determination of particle size distribution. Sieving method. British Standard Institution, London, UK, 2012.

[25] British Standards Institutions BS EN 16637-2:2023 - Construction products: Assessment of release of dangerous substances Horizontal dynamic surface leaching test, British Standard Institution, London, UK, 2023.

[26] Mehta, P. K. & Monteiro, P. J. M. *Concrete: Microstructure, properties, and materials* 4th edn. (McGraw-Hill, 2014).

[27] Istuque, D. B. et al. Impedance spectroscopy as a methodology to evaluate the reactivity of metakaolin based geopolymers. *Materials* **15**(23), 8387 (2022). 36499884 10.3390/ma15238387 PMC9740586

[28] Sun, Z. & Vollpracht, A. Leaching of monolithic geopolymer mortars. *Cem. Concr. Res.* **136**, 106161 (2020).

[29] Deutsches Institut für Bautechnik (DIBt). *Muster-Verwaltungsvorschrift Technische Baubestimmungen (MVVTB), Appendix 10 (in German)*, Berlin (2017).

[30] Dijkstra, J.J., Van der Sloot G. H.A., 2013. Dossier of information justifying that concrete qualifies for the potential release of regulated dangerous substances without- further-testing (WFT) by the Producer, ECN-X-13-008.

[31] Santhanam, M., Cohen, M. D. & Olek, J. Mechanism of sulfate attack: A fresh look Part 1: Summary of experimental results. *Cem. Concr. Res.* **32**(6), 915–921 (2002).

[32] Kosson, D. S., van der Sloot, H. A., Sanchez, F. & Garrabrants, A. C. An integrated framework for evaluating leaching in waste management and utilization of secondary materials. *Environ. Eng. Sci.* **19**(3), 159–204 (2002).

[33] Van Deventer, J. S. J., Provis, J. L. & Duxson, P. Technical and commercial progress in the adoption of geopolymer cement. *Miner. Eng.* **29**, 89–104 (2012).

[34] Lloyd, N., & Rangan, B. V. Geopolymer concrete: A review of development and opportunities. In *Proceedings of the 35th Conference on Our World in Concrete & Structures*, (2010).

[35] Van der Sloot, H.A. & Dijkstra, J.J. Development of horizontally standardized leaching tests for construction materials: a material or release based approach? Identical leaching mechanisms for different materials. *Report* ECN-C--04-060. (2004) 10.13140/RG.2.2.11986.76486.

[36] Liu, Q. et al. Heavy metal leaching behaviour and long-term environmental risk assessment of cement-solidified municipal solid waste incineration fly ash in sanitary landfill. *Chemosphere* **298**, 134571 (2022). 10.1016/j.chemosphere.2022.134571 35413369

[37] Belevi, H. & Baccini, P. Long-term behavior of municipal solid waste landfills. *Waste Manag. Res.* **7**(1), 43–56 (1989).

[38] Diotti, A., Perèz Galvin, A., Piccinali, A., Plizzari, G. & Sorlini, S. Chemical and leaching behavior of construction and demolition wastes and recycled aggregates. *Constr. Build. Mater.* **258**, 119548 (2020).

[39] Celis-Hernandez, O., Cundy, A. B., Croudace, I. W. & Ward, R. D. Environmental risk of trace metals and metalloids in estuarine sediments: An example from Southampton Water, UK. *Mar. Pollut. Bull.* **178**, 113580 (2022). 35366548 10.1016/j.marpolbul.2022.113580

[40] Santana-Casiano, J. M., González-Dávila, M. & Millero, F. J. The role of Fe(II) species on the oxidation of Fe(II) in natural waters in the presence of O_2_ and H_2_O_2_. *Mar. Chem.* **99**(1–4), 70–82 (2006).

[41] Shaked, Y., Twining, B. S., Browning, T. J., Koedooder, C. & Kranzler, C. F. Trace metal biogeochemistry in the ocean: From chemical principles to biological complexity. In *Treatise on Geochemistry* 3rd edn, Vol. 4 (eds Anbar, A. D. & Weis, D.) 371–414 (Elsevier, Amsterdam, 2024).

[42] Stumm, W. & Morgan, J. J. *Aquatic chemistry: Chemical equilibria and rates in natural waters* (John Wiley & Sons, 1996).

