# Supplementary material for: Environmental safety of electronic and construction and demolition waste based geopolymer breakwaters under simulated marine leaching
Source: Sci Rep. 2026 Jun 23;16:28655. doi: 10.1038/s41598-026-58190-x (PMC13575179; doi:10.1038/s41598-026-58190-x)
Supplement: Supplementary file 1 [file 41598_2026_58190_MOESM1_ESM.docx]

**SUPPORTING MATERIAL**

**Systematic experimental investigation of environmental aspects of release of metal ions to marine environment by waste based geopolymer breakwaters**

K.K.D.A. Wijesekara ^1^, Iacopo Carnacina^1*^, Monower Sadique ^1^, U. Dave^2^, A.M.M. Al Bakri^3^

* Corresponding author

Iacopo Carnacina - i.carnacina@ljmu.ac.uk

1 Built Environment and Sustainable Technologies (BEST) Research Institute, School of Civil Engineering and Built Environment, Liverpool John Moores University, Byrom Street, Liverpool, L3 3AF, UK
2 Department of Civil Engineering, Nirma University, S G Highway, Ahmedabad, 382481, Gujarat, India
3 Faculty of Chemical Engineering & Technology, Universiti Malaysia Perlis (UniMAP)  P.O. Box 77, D/A Pejabat Pos Besar 01000 Kangar, Perlis, Malaysia

Table S1 Concentrations of each metal ion released to DIW and calculations to determine their release mechanism

Concentrations of each metal ion released to SW and calculations to determine their release mechanism

British Standard Institution (2008). "BS EN 12620:2002+A1:2008. Aggregates for concrete."

British Standard Institution (2011). "BS EN 197-1: 2011. Cement. Part 1: Composition, specifications and conformity criteria for common cements."

British Standards Institutions (2012). BS EN 933-1:2012 Tests for geometrical properties of aggregates. Determination of particle size distribution. Sieving method. Brussels, BSI, European Committee for Standardization (CEN), Brussels.
